# Longitudinal home-cage automated assessment of climbing behavior shows sexual dimorphism and aging-related decrease in C57BL/6J healthy mice and allows early detection of motor impairment in the N171-82Q mouse model of Huntington’s disease

**DOI:** 10.3389/fnbeh.2023.1148172

**Published:** 2023-03-22

**Authors:** Rasneer S. Bains, Hamish Forrest, Rowland R. Sillito, J. Douglas Armstrong, Michelle Stewart, Patrick M. Nolan, Sara E. Wells

**Affiliations:** ^1^Mary Lyon Centre at Medical Research Council, Harwell, Oxfordshire, United Kingdom; ^2^Actual Analytics Ltd., Edinburgh, United Kingdom; ^3^School of Informatics, University of Edinburgh, Edinburgh, United Kingdom; ^4^Medical Research Council, Harwell Science Campus, Oxford, United Kingdom

**Keywords:** automated, neurodegeneration, motor function, reproducible, welfare, Huntington’s disease (HD), digital biomarkers

## Abstract

Monitoring the activity of mice within their home cage is proving to be a powerful tool for revealing subtle and early-onset phenotypes in mouse models. Video-tracking, in particular, lends itself to automated machine-learning technologies that have the potential to improve the manual annotations carried out by humans. This type of recording and analysis is particularly powerful in objective phenotyping, monitoring behaviors with no experimenter intervention. Automated home-cage testing allows the recording of non-evoked voluntary behaviors, which do not require any contact with the animal or exposure to specialist equipment. By avoiding stress deriving from handling, this approach, on the one hand, increases the welfare of experimental animals and, on the other hand, increases the reliability of results excluding confounding effects of stress on behavior. In this study, we show that the monitoring of climbing on the wire cage lid of a standard individually ventilated cage (IVC) yields reproducible data reflecting complex phenotypes of individual mouse inbred strains and of a widely used model of neurodegeneration, the N171-82Q mouse model of Huntington’s disease (HD). Measurements in the home-cage environment allowed for the collection of comprehensive motor activity data, which revealed sexual dimorphism, daily biphasic changes, and aging-related decrease in healthy C57BL/6J mice. Furthermore, home-cage recording of climbing allowed early detection of motor impairment in the N171-82Q HD mouse model. Integrating cage-floor activity with cage-lid activity (climbing) has the potential to greatly enhance the characterization of mouse strains, detecting early and subtle signs of disease and increasing reproducibility in preclinical studies.

## 1. Introduction

Advances in the field of genetics mean that mouse models are increasingly sophisticated, and more closely than ever before the model human disease (Mingrone et al., 2020). For diseases affecting neuromuscular systems, there is also an increasing range of assays and tests for mice that measure parameters such as coordination and muscle strength (Mandillo et al., 2014). Disorders of the central nervous system are often accompanied by deficits in motor function, and affect a number of aspects of movement, from locomotion and balance to finer tasks such as reaching and grasping (Tucci et al., 2007; Preisig et al., 2016). Climbing on the cage top and locomotor activity on the cage-floor have been found to be important indicators of motor function and form the natural activity routine of mouse motor behavior (Nevison et al., 1999; Borbélyová et al., 2019).

Currently, a number of tests, including grip strength and gait analysis (Tucci et al., 2007; Preisig et al., 2016), are used to study the progression of degenerative diseases. These are limited to measuring aspects of motor function, which can also be dependent on external factors, such as experimenter expertise, the timing of the test, testing conditions, and the motivation of the test subject (Balzani et al., 2018; Baran et al., 2022). In addition, a number of these tests are known to be affected by subtle factors, such as the order of testing and the amount of handling before testing, and crucially, repeated testing in progressive conditions may itself alter the results of subsequent tests (McIlwain et al., 2001; Paylor et al., 2006; Mingrone et al., 2020). Therefore, issues of reproducibility and consistency have to be overcome as researchers strive for greater translatability in preclinical research. This is particularly true for pharmacological investigations that require chronic administration of substances, especially as the short periods of time and potential external confounders affect the integrity and completeness of the result. The probability of clinical success for substances tested in such studies is therefore reduced (Kaffman et al., 2019).

Investigating perturbations in the home-cage activity of undisturbed mice over extended periods can greatly enrich standard out-of-cage phenotyping and provide novel insights into subtle and progressive conditions at early time points. A number of systems have been developed to investigate motor activity over extended periods of time in single-housed as well as group-housed mice. However, measuring both cage-lid climbing and cage-floor movement simultaneously in group-housed mice remained a technical challenge (Bains et al., 2018).

Over the past few years, there has been a concerted effort toward overcoming these challenges by housing the mice in testing chambers for extended periods of time and automatically measuring non-evoked activity (Bains et al., 2018). Voluntary wheel running has proved to be a robust indicator of motor-function deficits from an early stage, as it measures a number of motor parameters over several weeks (Lana-Elola et al., 2021). However, concerns such as single housing and the detrimental effect of exercise on certain genetically altered mouse strains that serve as models for diseases such as Huntington’s disease (HD; Corrochano et al., 2018), including the model used in the current study, in which wheel running may itself affect the phenotype expression, remain an issue. In addition, the subtler indicators of changes in motor function, such as activity around anticipation to light phase change, are not identified through wheel-running activity (Bains et al., 2016).

Despite some early promise shown by gene-targeting therapies, there is currently no disease-modifying treatment for HD, and therapy is focused on the management of symptoms and improving quality of life (Kim et al., 2021; Kwon, 2021). Progressive loss of neurons is a characteristic feature of this neurodegenerative condition. HD is caused by an unstable expansion within the trinucleotide poly(CAG) tract in exon 1 of the huntingtin gene, located on the short arm of Chromosome 4 (Menalled et al., 2012; Corrochano et al., 2014; Cepeda and Tong, 2018), and is characterized by progressive motor deficits such as loss of coordination, tremors, and hypokinesis (Schilling et al., 1999). The hallmark histopathology of HD is cell death in the striatum and cerebral cortex, which results in miscommunication between the basal ganglia and the cerebral cortex. This manifests as uncontrolled, involuntary movements (chorea), cognitive deficits, and psychiatric symptoms (Cepeda and Tong, 2018). The condition is a progressive, ultimately fatal, neurodegenerative disorder.

This study used the B6-TgN(HD82Gln)81Dbo/H, also known as the N171-82Q, model of HD, first published in 1999, in which damage to the basal ganglia structures causes a hyperkinetic disorder (chorea) in combination with a loss of voluntary movements (bradykinesia and rigidity). These phenotypes become evident at around 10.5 weeks of age and manifest as abnormal gait and other behavioral and physiological abnormalities, such as lower grip strength, disturbed limb dynamics, and rigidity of the trunk, as well as a tendency toward a lower body weight (Schilling et al., 1999; Ferrante, 2009; Preisig et al., 2016). Automated analysis of gait in the lateral and ventral plane has proved to be very useful in the early detection of the subtle changes in limb movement that recapitulate the hyperkinetic phenotype observed in this model, which is detected as early as 10.5 weeks of age (Preisig et al., 2016).

Voluntary locomotion in mouse disease models is highly clinically relevant because it provides an insight into the physiology of the condition, as well as the behavioral motivation of the individual, and is a fundamental readout of the phenotype used in the diagnosis in human patients (Karl Kieburtz et al., 1996; Reilmann et al., 2014). A method that measures the ways in which animals move in non-provoked situations could potentially be a powerful tool for detecting early, and complex, temporal phenotypes.

Advanced image analysis, which can highlight changes in the animal’s gait in both the lateral and the ventral plane, has thus far proven to be the most sophisticated way of extracting subtle motor phenotypes earlier than 13 weeks of age in the model used in our study (Preisig et al., 2016).

In this study, we demonstrate a new tool for the automated analysis of motor activity, which encompasses climbing as well as locomotion on the cage-floor, in undisturbed mice over multiple light: dark cycles. Through its application to the N171-82Q model of HD, we have uncovered a robust complex phenotypic profile for disease progression, including early features of motor dysfunction that are fundamental in developing reproducible digital biomarkers for therapeutic testing, especially when targeting the prodromal stages of HD.

In meeting these challenges, we developed an algorithm to automatically annotate climbing behavior from high-definition video captured from a side-on view of the home-cage. The resulting automated climbing behavior annotations provide an important additional parameter set that further enriches the activity profile captured by the Home Cage Analyser system (HCA; Actual Analytics Ltd., UK; Bains et al., 2016). Our study shows that it is possible to measure two robust indicators of activity simultaneously (cage-floor activity and cage-lid activity) in group-housed mice from the inbred strain C57BL/6J. Using this technology, we have also demonstrated the advantages of using a more comprehensive recording of motor activity to reveal early signs of degeneration in a genetically altered mouse model of HD (N171-82Q; Schilling et al., 1999).

## 2. Materials and methods

### 2.1. Animals and husbandry

All mice used in the study were bred in the Mary Lyon Centre at MRC Harwell and were housed in individually ventilated cages (IVCs; Tecniplast BlueLine 1284) in groups of three mice per cage on Eco-pure spruce chips grade 6 bedding (Datesand, UK), with shredded article shaving nesting material and small cardboard play tunnels for enrichment. The mice were kept under controlled light (light 07:00–19:00; dark 19:00–07:00), temperature (22°C ± 2°C), and humidity (55% ± 10%) conditions. They had free access to water (25 p.p.m. chlorine) and were fed *ad libitum* on a commercial diet [SDS Rat and Mouse No.3 Breeding diet (RM3)]. All procedures and animal studies were carried out in accordance with the Animals (Scientific Procedures) Act 1986, UK, Amendment Regulations 2012 (SI 4 2012/3039).

For the first study, 18 male and 18 female mice, in six cages of three mice each, from the inbred strain C57BL/6J were recorded at three time points: 13–14 weeks, 30–31 weeks, and 52–53 weeks of age. For the second study, mice from the mutant strain B6-TgN (HD82Gln) 81Dbo/H (HD), were recorded at three time points: 8 weeks, 13 weeks, and 15–16 weeks of age. Twenty-seven hemizygous (Hemi) males carrying the HD transgene, along with 24 male wild type (WT) littermate controls, and 33 hemizygous females carrying the HD transgene, along with 24 female WT mice, were housed in same-genotype groups of three mice per cage. Using the cage as the experimental unit, a sample number of six was calculated to be the most appropriate sample size based on data from previous studies (Supplementary Dataset 1). Therefore, the first study of C57BL/6J mice, had *n* = 6 for both sexes. Additional animals were included in the HD study to compensate for the high attrition rate experienced with this model. Therefore, 9–11 cages of hemizygous mice were included in the study to avoid under-powering the later time points (Supplementary Table 1, Supplementary Dataset 2). Data from all cages were included in the analysis and appropriate statistical methods (described below) were used to account for the differences in the group sizes. Mice were housed with colony-mates born within the same week in cages containing animals of the same genotype.

Three days prior to recording sessions, the animals were transferred to clean home cages with fresh bedding, nesting material, and a cardboard rodent tunnel as enrichment material, in line with the standard husbandry procedures for IVC cages. The cages were then placed in an IVC rack in the experimental room for the animals to acclimatize. For each recording, the cages were randomly assigned to an HCA rig. On the first day of recording, each cage was placed onto the ventilation system, within the rig, as would occur during a normal husbandry procedure.

Animal welfare checks were carried out visually twice daily. At the end of the recording period, the home cages were removed from the HCA rigs and returned to their original positions on the IVC racks.

### 2.2. Microchipping and data collection

Radio frequency identification microchips were injected subcutaneously into the lower left or right quadrant of the abdomen of each mouse at 12 weeks of age for the C57BL/6J study and 7 weeks of age for the B6-TgN(HD82Gln)81Dbo/H study. These microchips were contained in standard ISO-biocompatible glass capsules (11.5 × 2 mm; PeddyMark Ltd., UK). The procedure was performed on sedated mice (Isoflo; Abbott, UK) after topical application of local anesthetic cream on the injection site prior to the procedure (EMLA Cream 5%; AstraZeneca, UK) as described in Hobson et al. (2020). The animals were allowed to recover from the microchip procedure for at least 1 week before being placed in the HCA rigs for data collection. The procedure for data collection has been described previously in Bains et al. (2016) and Hobson et al. (2020), briefly, microchipped animals were placed in the HCA rigs for 72 h of continuous recording for every age group. At the end of the 72-h recording period for that age, the animals in their IVCs were returned to the standard IVC rack, until the animals reached the next age group to be recorded, where the procedure was repeated for another 72 h.

### 2.3. Measurement of climbing and validation

Mice were housed in an individually ventilated cage which has a metal wire lid that allows climbing. Mouse activity was videorecorded through a high definition, infra-red camera of the Home Cage Analyser system (HCA; Actual Analytics Ltd., UK), and videos were subsequently analyzed offline through a video-recognition algorithm. Through this method, climbing behavior is measured on a frame-by-frame basis by numerically characterizing the pattern of motion occurring within a pre-defined region around the cage lid and quantifying its similarity to a set of key reference examples of climbing and non-climbing behavior (selected programmatically from a large set of human "training" annotations) to yield a classification decision. More specifically, the local trinary pattern representation proposed by Yeffet and Wolf (2009), is used to characterize motion within a 690 × 385 pixel region adjacent to the cage lid as a 16,384-dimensional vector; this particular representation was shown in Burgos-Artizzu et al. (2012) to provide an effective, yet computationally efficient, means of distinguishing between different mouse behaviors. Local trinary pattern vectors were extracted for every video frame across more than 7 h of human-annotated video footage—encompassing over 130 separate bouts of climbing—and were used to train a linear support vector machine classifier (SVM; Fan et al., 2008) to distinguish between climbing and non-climbing instances. To leverage the correlation between consecutive video frames, a temporal voting window was applied, such that the final classification of a given video frame represented the consensus over a wider time period spanning the frame in question (the underlying logic is to reduce spurious “single frame” detections, while conversely preventing erroneous “splitting” of longer bouts of climbing on the basis of a single misclassified frame.) A leave-one-out cross-validation procedure was applied over the available “training” set of 15 discrete 30-min video segments, in order to identify: (i) the SVM regularization parameter values; and (ii) the temporal aggregation parameters that—on average—yielded the best generalization performance. The final classifier, generated from the full set of available training data using the parameters revealed by the preceding cross-validation process, was then tested on a further 2.5 h of—unseen—annotated test videos. These videos were scored by three separate annotators, whose consensus was derived using a “majority voting” rule, to yield a single “gold standard” annotation for each video: comparison to this gold standard annotation yielded 86.6% frame-by-frame accuracy (where 76.9% of climbing frames and 89.2% of non-climbing frames were correctly classified, with climbing behavior accounting for approximately 21% of the test data). Considering this test data in terms of 5-min time bins, automatically annotated climbing time correlates well with human annotated climbing time, as confirmed by Spearman’s rank coefficient (*ρ* = 0.862; *n* = 30; *p* < 0.000000001).

It is however worth noting that there are other ways to combine the human annotations. For example, during training, a frame was treated as climbing if any one annotator deemed it so (the purpose of this was to ensure that no example of climbing was missed). If the test annotations are combined in this way, a greater proportion of the data is considered to be climbing (approximately 30%), and the accuracy scores of the automated climbing annotations change accordingly (85.6% frame-by-frame accuracy with 65.9% of climbing frames, and 94.3% of non-climbing frames correctly classified). Correlation over 5 min time bins is lower, albeit broadly similar (*ρ* = 0.836; *n* = 30; *p* < 0.00000001).

### 2.4. Data analysis

#### 2.4.1. Linear mixed-effects modelling

To account for dependence between data recorded over separate days from the same cage (repeated measures) and to avoid pseudo-replication, statistical analyses were conducted using linear mixed-effects modelling. To adjust for parameters with non-normal distributions, data were box-cox transformed prior to analysis. Any subsequent modelling satisfied assumptions of normally distributed residuals.

We constructed a linear mixed-effects model of either distance travelled or time spent climbing (continuous variables) as a function of the effect of sex, age of caged mice, and genotype (all categorical fixed effects). Cage ID was modelled as the random effect intercept with day of recording as the random effect slope. This structure allows for cages to vary randomly in their baseline distance moved or time spent climbing value, and for this relationship to vary randomly according to the day of recording. It will account for time-dependent and cage-specific fluctuations in activity over the 3 days of recording.


$$Mean\;activity∼Age:Genotype:Sex+\left(day\;of\;recording|Cage\;ID\right)$$

This model was compared to other model iterations with different combinations of sex, age, and genotype with or without the interaction term and random effects structure. An ANOVA test was run to determine the statistical significance of the interaction between Age: Genotype: Sex and to inform model selection. Random effects and fixed effects found not to have a statistically significant contribution to model fit were eliminated. Models were fit using R’s “lmer” function.

#### 2.4.2. Time-frames of interest

The time-frames of interest in the current study were defined as the 30 min directly preceding lights being turned on (06:30–07:00) and 30 min directly preceding lights being turned off (18:30–19:00). This definition was consistent between both parameters of interest: distance travelled (mm) and time spent climbing (seconds). When analyzing activity during the time-frames of interest, we first summed data per time bin (6 min) for the mouse within a cage for distance travelled and cumulative climbing in the cage per time bin (6 min) for time spent climbing. We then calculated the average activity per cage across the 5 time bins.

One of the early indicators in clinical presentation of a number of neurodegenerative conditions is a perturbed circadian rhythm (Carter et al., 2021). We have previously shown that C57BL/6J mice show peak activity in the dark phase, and that mouse activity varies around a change in light phases in a strain-specific manner (Bains et al., 2016). In the current study, these changes were particularly relevant, as mouse models of neurodegenerative diseases, including HD, have known sleep disturbances, that manifest as changes to the time of onset and/or offset of the active periods (Werdann and Zhang, 2020). Sleep onset latency towards the end of the active period is a particularly sensitive measure (Morton et al., 2005), therefore the first 30 min and the last 30 min of the active period were chosen as the time frames of interest for further investigation.

#### 2.4.3. *Post-hoc* analysis

We conducted pairwise *post-hoc* comparison tests by computing the estimated marginal means (least-squares means) for factor combinations and correcting for multiple comparisons using the Benjamini–Hochberg method to decrease the false discovery rate. This process was run using R’s “emmeans” function, which returned adjusted p values. These values were used to indicate the statistical significance of the genotype effect at various levels of factor combination.

While the algorithm for automated behavior annotation is proprietary, the analysis is openly available as a part of this manuscript; please see “Data availability statement” in this manuscript. The datasets for the experiments in this manuscript are also openly available on request.

All climbing data were converted from a number of frames to time spent climbing in seconds prior to analysis and visualization, as the authors believe that to be a more intuitive parameter. The number of frames is converted to time in seconds as follows:


$$\text{Time Spent Climbing in Seconds=}\left(\text{Number of frames*40}\right)\text{/1,000}$$

Each individual frame is 40 ms long.

## 3. Results

### 3.1. Multiday recording in mouse home cage shows sexual dimorphism in C57BL/6J mice and reveals age-related decrease in activity

**Figure 1:**
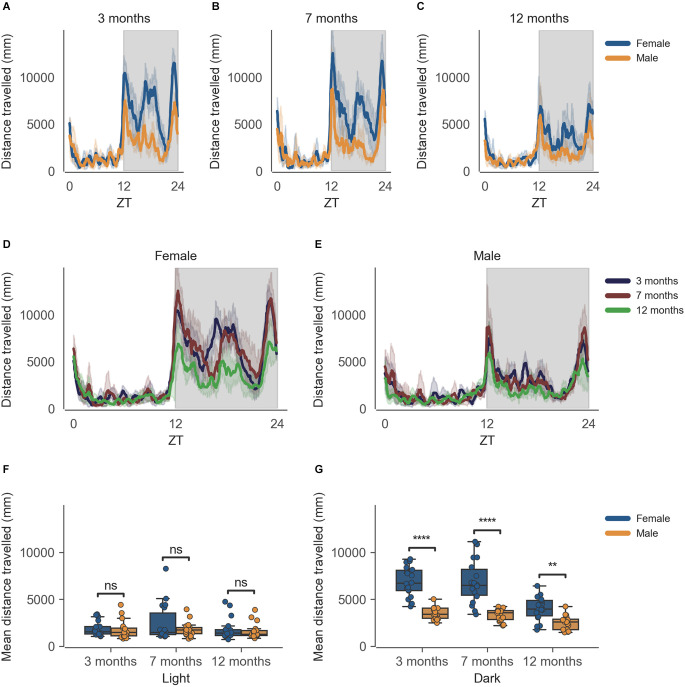
Distance travelled by mice during the dark phase varies according to sex and across age during passive home-cage monitoring. **(A)** Distance travelled (mm) over zeitgeber time in female and male cages of 3-month-old mice during the recording session, binned into 6-min time bins and averaged over a 24-h period. The line represents the mean distance over time across cages of a sex group; the shaded error band represents a 95% confidence interval. Data from individual mice within a cage were summed to produce one time series per cage. The gray shaded areas on the plot represent darkness. **(B,C)** Same as **(A)** but for 7-month-old and 12-month-old mice, respectively. **(D)** Distance travelled over zeitgeber time in female cages of mice of all ages. The line represents the mean distance over time across cages of a sex and age group; the error-shaded area represents 95% confidence interval. **(E)** Same as **(D)** but for male cages. **(F)** Boxplot of mean distance travelled during light phase with cages split by age and by sex. Distance travelled within the light phase was averaged across time per cage and per day. Three data points per cage (3 days of recording) were modelled using a linear mixed-effects model to account for repeated-measures and least-squares means estimated to return adjusted p values of levels of factor combinations. **(G)** Same as **(D)** but for the dark phase. ns *p* > 0.05, **p < 0.05, ****p < 0.0001.

The data from the females of inbred strain C57BL/6J show significantly higher cage-floor activity, described as distance travelled in mm (Figure 1G) in the dark phase, as compared to males at 3 months of age (*n* = 6, *p* < 0.0001). This difference in cage-floor activity during the dark phase persists as the mice age, as seen in the later time points of 7 months (*p* = 0.0001) and 12 months (*p* < 0.01; Figure 1G, *n* = 6). The activity shows a clear circadian rhythm (Figure 1). Individual comparisons between each age group, per sex, also reveal statistically significant differences in the dark phase for both females and males at 12 months of age as compared to 3 months and 7 months of age (Supplementary Figure 1, Supplementary Dataset 2).

### 3.2. Automated climbing annotation in mouse home cage records complex sexual dimorphism in C57BL/6J mice and reveals age-related decrease in activity

**Figure 2:**
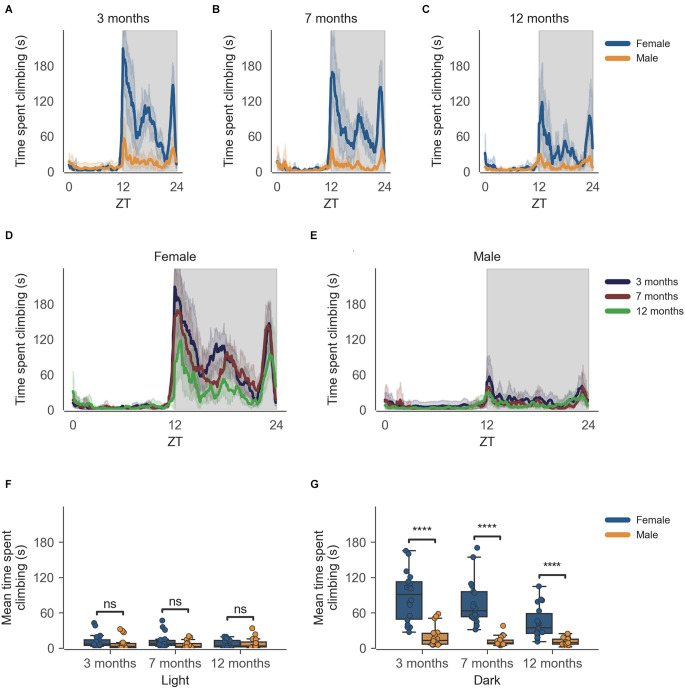
Time spent climbing by mice during dark phase varies according to sex and across age during passive home-cage monitoring. **(A)** Time spent climbing over zeitgeber time in female and male cages of 3-month-old mice during recording session, binned into 6-min time bins and averaged over a 24-h period. Line represents mean time spent climbing over time across cages of a sex group; the shaded error band represents a 95% confidence interval. Data from individual mice within a cage were summed to produce one time series per cage. The gray shaded areas on the plot represent darkness. **(B,C)** Same as **(A)** but for 7-month-old and 12-month-old mice, respectively. **(D)** The time spent climbing over zeitgeber time in female cages of mice of all ages. The line represents the mean distance over time across cages of a sex and age group; the error-shaded area represents a 95% confidence interval. **(E)** Same as **(D)** but for male cages. **(F)** Boxplot of mean time spent climbing moved during light phase with cages split by age and by sex. Time spent climbing within the light phase was averaged across time per cage and per day. Three data points per cage (3 days of recording) were modelled using a linear mixed-effects model to account for repeated-measures and least-squares means estimated to return adjusted p values of levels of factor combinations. **(G)** Same as **(D)** but for the dark phase. ns *p* > 0.05, ****p < 0.0001.

The data from the females of inbred strain C57BL/6J show significantly higher cage-lid climbing, described as time spent climbing in seconds (Figure 2), as compared to males at 3 months of age (Figure 2G, *n* = 6, *p* < 0.0001), this difference in cage-lid climbing during the dark phase persists as the mice age as seen at the later time points of 7 months (Figure 2G, *n* = 6, *p* < 0.0001) and 12 months (Figure 2G, *n* = 6, *p* < 0.0001). The activity shows a clear circadian rhythm (Figure 2). Individual comparisons between each age group per sex reveal statistically significant differences in the dark phase for females only, at 12 months of age as compared to 3 months and 7 months of age (Supplementary Figure 2, Supplementary Dataset 2).

### 3.3. Early detection of activity phenotype in mouse model of Huntington’s disease

**Figure 3:**
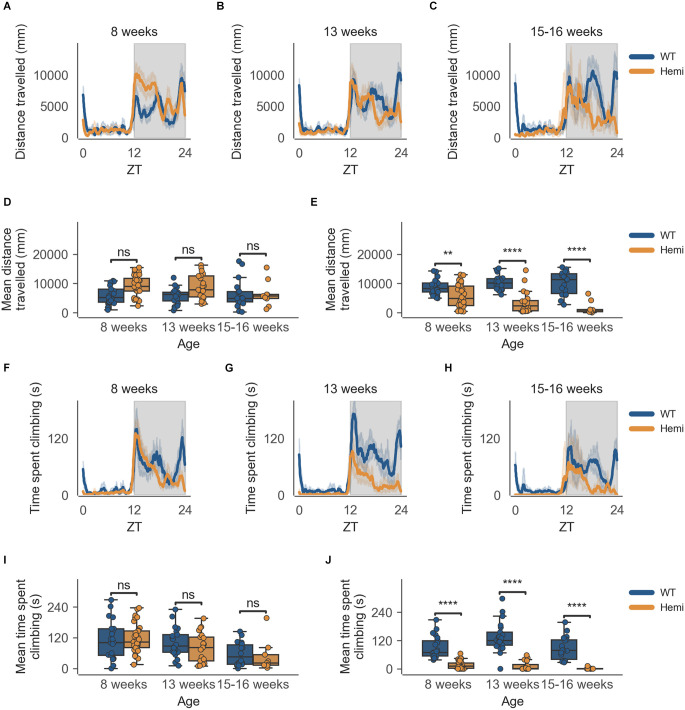
Distance travelled and climbing activity varies according to genotype in female mice and across age at the conclusion of the dark phase, but not at beginning of the dark phase. **(A)** Distance travelled over zeitgeber time in female-only cages of 8-week-old mice, split according to genotype, during the recording session, binned into 6-min time bins and averaged over a 24-h period. The line represents the mean distance over time across cages of a genotype group, the shaded error band represents a 95% confidence interval. Data from individual mice within a cage was summed to produce one time-series per cage. The gray shaded areas on the plot represent darkness. **(B,C)** Same as **(A)** but for 13-week-old and 15–16-week-old mice, respectively. **(D)** Boxplot of mean distance travelled during first 30 min of darkness within female-only cages split by age and genotype. Distance travelled was averaged across the first 30 min of darkness per cage and per day. Three data points per cage (3 days of recording) were modelled using a linear mixed-effects model to account for repeated-measures and least-squares means estimated to return adjusted p values of levels of factor combinations. **(E)** Same as **(D)** but for last 30 min of darkness. **(F)** Time spent climbing over zeitgeber time in female-only cages of 8-week-old mice, split according to genotype, during the recording session, binned into 6-min time bins and averaged over a 24-h period. The line represents the mean time spent climbing over time across cages of a genotype group, the shaded error band represents a 95% confidence interval. Data from individual mice within a cage was summed to produce one time-series per cage. The gray shaded areas on the plot represent darkness. **(G,H)** Same as **(F)** but for 13-week-old and 15–16-week-old mice, respectively. **(I)** Boxplot of mean time spent climbing during first 30 min of darkness within female-only cages split by age and genotype. Time spent climbing was averaged across the first 30 min of darkness per cage and per day. Three data points per cage (3 days of recording) were modelled using a linear mixed-effects model to account for repeated-measures and least-squares means estimated to return adjusted p values of levels of factor combinations. **(J)** Same as **(I)** but for last 30 min of darkness. ns *p* > 0.05, **p < 0.01, ****p < 0.0001.

**Figure 4:**
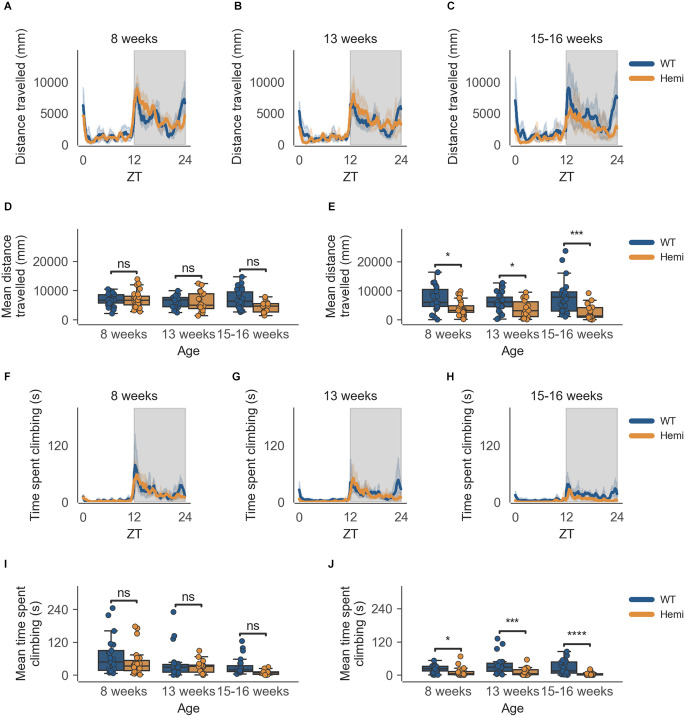
Distance travelled and climbing activity varies according to genotype in male mice and across age at conclusion of dark phase, but not at beginning of dark phase. **(A)** Distance travelled over zeitgeber time in male-only cages of 8-week-old mice, split according to genotype, during the recording session, binned into 6-min time bins and averaged over a 24-h period. Line represents mean distance over time across cages of a genotype group, the shaded error band represents a 95% confidence interval. Data from individual mice within a cage was summed to produce one time-series per cage. The gray shaded areas on the plot represent darkness. **(B,C)** Same as **(A)** but for 13-week-old and 15–16-week-old mice, respectively. **(D)** Boxplot of mean distance travelled during first 30 min of darkness within male-only cages split by age and genotype. Distance travelled was averaged across the first 30 min of darkness per cage and per day. Three data points per cage (3 days of recording) were modelled using a linear mixed-effects model to account for repeated-measures and least-squares means estimated to return adjusted p values of levels of factor combinations. **(E)** Same as **(D)** but for last 30 min of darkness. **(F)** Time spent climbing over zeitgeber time in male-only cages of 8-week-old mice, split according to genotype, during the recording session, binned into 6-min time bins and averaged over a 24-h period. The line represents the mean time spent climbing over time across cages of a genotype group, the shaded error band represents a 95% confidence interval. Data from individual mice within a cage was summed to produce one time-series per cage. The gray shaded areas on the plot represent darkness. **(G,H)** Same as **(F)** but for 13-week-old and 15–16-week-old mice, respectively. **(I)** Boxplot of mean time spent climbing during first 30 min of darkness within male-only cages split by age and genotype. Time spent climbing was averaged across the first 30 min of darkness per cage and per day. Three data points per cage (3 days of recording) were modelled using a linear mixed-effects model to account for repeated-measures and least-squares means estimated to return adjusted p values of levels of factor combinations. **(J)** Same as **(I)** but for last 30 min of darkness. ns *p* > 0.05, *p < 0.05, ***p < 0.001, ****p < 0.0001.

The data from the HD model recapitulate the clear circadian rhythm and sex differences seen in the inbred strain, in which females were significantly more active than males in both measures of activity (cage-floor activity, as seen in Figures 3A–C and Figures 4A–C and cage-lid climbing activity, as seen in Figures 3F–H and Figures 4F–H). The total activity over the dark and light phases of hemizygous (Hemi) HD mice compared to the WT HD mice was not statistically different from each other for both sexes.

There is, however, a specific time-of-day-dependent deficit in activity seen in Hemi HD mice as compared to WT HD mice, at the transition between dark and light phases for females, not seen in the transition between light and dark phases (18:30–19:00; Figure 3D). This difference was apparent as early as 8 weeks when the animals showed no overt signs of the disease onset (Figure 3E, *n* = 8 WT/11 Hemi, *p* < 0.01). The decrease in cage-floor activity at this time became even more apparent at 13 weeks of age (Figure 3E, *n* = 7 WT/7 Hemi, *p* < 0.0001). By 15–16 weeks of age the mice showed clear signs of disease and differences between the two genotypes were distinct (Figure 3E, *n* = 7 WT/3 Hemi, *p* < 0.0001).

This decrease in activity at the end of the dark phase (06:30–07:00) is also seen in male Hemi HD mice as compared to WT HD mice, allowing for the phenotype to be detected as early as 8 weeks of age (Figure 4E, *n* = 8 WT/9 Hemi, *p* < 0.05), persisting into the next time point of 13 weeks of age (Figure 4E, *n* = 8 WT/6 Hemi, *p* < 0.05), and becoming obvious at 15–16 weeks of age (Figure 4E, *n* = 8 WT/5 Hemi, *p* < 0.001). Once again this decrease in activity is not seen in the transition between light and dark phases (18:30–19:00; Figure 4D).

This specific time-of-day-dependent deficit in cage-floor activity is mirrored in cage-lid climbing, described as time spent climbing, where female Hemi HD mice show a significant decrease in time spent climbing compared to female WT HD mice, at the transition between dark and light phases (06:30–07:00), not seen in the transition between light and dark phases (18:30–19:00; Figure 3I). Once again, this difference was apparent as early as 8 weeks when the animals showed no overt signs of the disease onset (Figure 3J, *n* = 8 WT/11 Hemi, *p* < 0.0001). As seen with cage-floor activity, the decrease in cage-lid climbing at this time became even more apparent at 13 weeks of age (Figure 3J, *n* = 7 WT/7 Hemi, *p* < 0.0001) and by 15–16 weeks of age the mice showed clear signs of disease and differences between the two genotypes were distinct (Figure 3J, *n* = 7 WT/3 Hemi, *p* < 0.0001).

As with cage-floor activity, time spent climbing also follows the same pattern in male Hemi HD mice as compared to male WT HD mice, where a statistically significant difference in genotypes is observed at 8 weeks of age (Figure 4J, *n* = 8 WT/9 Hemi, *p* < 0.05), persisting to the next time point of 13 weeks of age (Figure 4J, *n* = 8 WT/6 Hemi, *p* < 0.001) and becoming obvious at 15–16 weeks of age (Figure 4J, *n* = 8 WT/5 Hemi, *p* = 0.0001). Again this decrease in activity is not seen in the transition between light and dark phases (18:30–19:00; Figure 4I).

## 4. Discussion

Classically, with a few exceptions, all behavior testing is carried out during the light phase, in which resting animals are removed from their home cage and placed in a novel environment away from their cage mates (Bains et al., 2018). Such out-of-cage tests are known to be influenced by factors such as ambient noise, lighting and odors, and handling methods, resulting in stress and anxiety-like responses. Home-cage monitoring, by contrast, is free of such influences as these behavioral confounds are removed. Therefore, the welfare burden on the animals is much lower. Avoidance of stressors deriving from out-of-cage testing, on the one hand, leads to a more animal-friendly behavioral testing and, on the other hand, increases the validity and reproducibility of results (Voikar and Gaburro, 2020; d’Isa and Gerlai, 2023). Two further advantages of home-cage testing are the possibility to test the animals in a social environment, while they are group-housed, and the possibility to record behaviors outside of the normal observation hours, allowing for continual monitoring of progressive phenotypes over both light and dark cycles and not just at specific time points. Phenotyping through tests lasting a few minutes performed during the conventional working hours of researchers is an approach that risks missing critical milestones for disease emergence and/or progression. This is particularly true for aged mice, as it has been shown that spontaneous locomotor activity declines with age (Nakamura et al., 2011; Yanai and Endo, 2021) and snapshots of these milestones may not be enough to reveal complex phenotypes that change with time of day, or are particularly exaggerated at certain times of the day in relation to the light: dark cycle, as shown in this study.

The advantage of observing the mice undisturbed within their home cage over multiple light:dark cycles is that, in addition to the observed phenotype, it is also possible to disentangle the temporal appearance of such phenotypic traits by focusing the analysis on specific time-frames of interest. The data from C57BL/6J show that there is a clear sexual dimorphism in the overall activity of the animals and that both males and females show peak activity in the dark phase. These data, therefore, point to a clear circadian influence. This method of analysis allows one to also investigate the influence of ultradian parameters on measures such as cage-floor activity and cage-lid climbing. The importance of understanding these circadian and ultradian effects is highlighted in the HD study, in which the males show very low baseline activity in both mutant and WT strains. We have already shown that the HCA system is capable of detecting statistically significant changes in activity around the light phase changes between various background strains (Bains et al., 2016). Here we extend this concept to draw out clinically relevant, subtle, and early phenotypic changes by focusing on specific times of interest such as the first 30 and last 30 min of the dark phase.

Through the current study, we showcase a recently developed automated behavior annotation tool for climbing behavior in standard IVCs under group-housed conditions. We have previously shown the capabilities of the system in investigating cage-floor activity in group-housed mice in their home cages (Bains et al., 2016). Here, we show that measuring climbing is part of the standard motor behavior repertoire of mice and can greatly enhance the existing dataset to investigate motor phenotypes in much greater detail and with minimal experimenter intervention.

Sexual dimorphism in climbing behavior has been reported previously in singly tested C57BL/6Ntac mice, using the LABORAS system, in which the main aim of the study was to investigate the difference in response to novelty between the sexes. Furthermore, parameters were only measured for 10 min (Borbélyová et al., 2019). More recently, a study on the effect of age, sex, and strain on cage-lid climbing in single-housed mice has also reported sexual dimorphism, as well as strain and age differences, in single-housed mice, over 24 h, peaking in the dark phase (Zhang et al., 2021). The data from the inbred strain C57BL/6J, in the current study show significantly high cage-lid climbing as well as cage-floor activity in females as compared to males at all three age time points, with most of the activity observed in the dark phase. To the best of our knowledge, this is the first system of its kind that can detect cage-level climbing activity in group-housed mice within their home cage for extended periods of time, without the need for removal into specialized and/or novel caging.

Serious motor and cognitive deficits that are the hallmark of HD are often preceded, by decades, by more subtle changes in circadian rhythms and motor function (Wang et al., 2018; Wiatr et al., 2018). Therefore, an approach that screens for the chronic and progressive nature of such conditions is more clinically relevant than one that screens for acute signs of motor deficits that manifest at a much later stage of the disease. One such approach is to focus on behaviors that are elective and not essential to survival, such as grooming, playing, or climbing, as they reflect the animal’s emotional or motivational state, which would be ethologically more relevant for a preclinical model (Zhang et al., 2021). Therefore, a perturbation in such behaviors could reflect a suboptimal health state, especially in conditions that are chronic and progressive rather than acute.

The onset of HD is often insidious and progressive and the phenotypes are biphasic; at early stages of the condition involuntary functions are affected and in the later stages the directly controlled, voluntary functions begin to fade. This means that motor phenotypes are often expressed as hyperkinesia in the early stages and akinesia in the later stage (Kim et al., 2021). Therefore, it becomes imperative to investigate such conditions longitudinally and for extended periods of time as the “snapshot in time” investigations, such as those that investigate motor activity in an open field, may not be representative of a clinically relevant disease profile.

The HD data in the current study show a significant increase in signal in both cage-floor and cage-lid climbing activities around the transition between the light-to-dark and dark-to-light phases. There is ample evidence to show that spontaneous cage-lid climbing is mediated through the dopaminergic system and therefore depends on the motivation and arousal state of the mouse (Costall et al., 1982; Palmiter, 2008; Brooks and Dunnett, 2009). As mice are active during the dark phase, the arousal states coincide with the transition periods between the light and dark phases of the circadian cycle; we have already shown that the most active periods as seen from cage-floor activity, are around these transition times (Bains et al., 2016). The current study shows that this is also true for cage-lid climbing. In addition, despite the decrease in total activity with age, this increase in cage-lid climbing and cage-floor activity around dark-to-light phase transition persists. This aspect is of particular interest in those models in which the genetic manipulation modelling the disease would result in a greater decrease in activity with age, as compared to wild-type counterparts. However, as the activity in the wild-type mice also decreases with age, any decrease in activity due to the genotype is therefore hard to discern in conventional testing paradigms.

The importance of this finding is highlighted in the set of experiments carried out using the mouse model of HD, N171-82Q. These data recapitulate the sex differences seen in the C57BL/6J strain experiments, in which females were significantly more active than males in both measures of activity, with this difference persisting across all time points. However, of note is a specific time-dependent decrease, compared with WT mice, in cage-lid climbing activity at the transition between dark and light phases, which was apparent as early as 8 weeks, when the animals showed no overt signs of the disease onset. The decrease in cage-floor activity at this time was also observed at 8 weeks of age; however, the decrease in cage-floor activity became even more apparent at 13 weeks of age. By 15–16 weeks of age the mice showed clear signs of disease and differences between the two genotypes were distinct. This last finding is of particular interest as clinical case studies, as well as mouse models of HD, are known to present with sleep disturbances, one of the hallmarks of the condition (Pallier et al., 2007). Whilst the mechanism is not fully understood, there is some evidence that this change may be attributed to increased pathology either in the brain region controlling circadian rhythms—the suprachiasmatic nucleus—or in a pathway further downstream (Pallier et al., 2007).

This study recapitulates the aspect of the disease in which the offset of activity in HD mice is observed sooner than that in their WT counterparts, for both cage-floor activity, as well as cage-lid climbing. Indeed, a 2005 study comparing the circadian activity patterns of human patients with a different mouse model of Huntington’s disease (R6/2), reported a similar pattern of decline in activity towards the end of the active phase with disease progression (Morton et al., 2005). In human patients, this manifests as spending longer time in bed. However, in the absence of a complete circadian screen, which would be outside the scope of this study, it would not be over-anthropomorphizing to say that HD mice begin their rest period earlier than their wild-type counterparts and remain at rest for longer, from the earliest stages of the disease.

In progressive degenerative conditions, neuronal dysfunction occurs before any overt signs of the condition become apparent in the behavior. As neurons are unable to regenerate, most therapies under development focus on neuroprotection, with the aim of slowing the progression of the disease and, where possible, delaying the onset (Jin et al., 2014; Kumar et al., 2015). This necessitates the development of models in which the therapeutic window aims to target the pre-manifestation period, in order to minimize neuronal loss. Therefore, any models that can identify the earliest manifestation of the mutation are invaluable in the investigation of the disease progression and identification of early biomarkers (Levine et al., 2004).

It is important to remember, however, that animal behavior is complex, and that external factors, such as the effects of diet and exercise, can have an impact on disease progression (Dutta et al., 2021). The model used in the current study, N171-82Q, is known to have a more variable phenotype than that of the more severe R6/2 model, even though the motor phenotype and weight loss generally becomes evident at 11 weeks of age (Ferrante, 2009). Such disease models are generally complex in their development, and part of the required improvement in animal research is the development of tools with the ability to capture this complexity both in terms of different phenotypes measured and the timings of their appearance. The factors driving spontaneous cage-lid climbing are not fully understood, but it is clear that this activity is affected by a decline in welfare (Zhang et al., 2021). Thus, we can say that investigating the total non-evoked motor function repertoire of animals in progressive and degenerative conditions is the first step toward early phenotype recognition and this approach can be extended to other mutant models showing complex phenotypes.

## Data availability statement

The datasets presented in this study can be found in online repositories. The names of the repository/repositories and accession number(s) can be found in the article/Supplementary material.

## Ethics statement

Animal studies described here were subject to the guidance issued by the Medical Research Council in Responsibility in the Use of Animals for Medical Research (July 1993), were dependent on an institutional Animal Welfare and Ethical Review Body evaluation and were carried out in compliance with the Animals (Scientific Procedures) Act 1986, UK, Amendment Regulations 2012 (SI 42012/3039).

## Author contributions

RB was responsible for the experimental design, experimental procedure, data collection and manuscript preparation. HF carried out bioinformatics and statistical analysis. RS was responsible for the system design, including automated climbing algorithm. JA contributed to the study design and system design. MS contributed to the study design. PN contributed to the study design, carried out circadian and activity data analysis, and prepared the manuscript. SW contributed to the study design, including animal procedures, and prepared the manuscript. All authors contributed to the article and approved the submitted version.

## References

[B1] Bains R. S. Cater H. L. Sillito R. R. Chartsias A. Sneddon D. Concas D.. (2016). Analysis of individual mouse activity in group housed animals of different inbred strains using a novel automated home cage analysis system. Front. Behav. Neurosci. 10, 1–12. 10.3389/fnbeh.2016.00106 27375446 PMC4901040

[B2] Bains R. S. Wells S. Sillito R. R. Armstrong J. D. Cater H. L. Banks G.. (2018). Assessing mouse behaviour throughout the light/dark cycle using automated in-cage analysis tools. J. Neurosci. Methods 300, 37–47. 10.1016/j.jneumeth.2017.04.014 28456660 PMC5909039

[B3] Balzani E. Falappa M. Balci F. Tucci V. (2018). An approach to monitoring home-cage behavior in mice that facilitates data sharing. Nat. Protoc. 13, 1331–1347. 10.1038/nprot.2018.031 29773907

[B4] Baran S. W. Bratcher N. Dennis J. Gaburro S. Karlsson E. M. Maguire S.. (2022). Emerging role of translational digital biomarkers within home cage monitoring technologies in preclinical drug discovery and development. Front. Behav. Neurosci. 15:758274. 10.3389/fnbeh.2021.758274 35242017 PMC8885444

[B5] Borbélyová V. Janišová K. Mysliveček J. Riljak V. (2019). Sex-related differences in locomotion and climbing of C57Bl/6NTac mice in a novel environment. Physiol. Res. 68, S353–S359. 10.33549/physiolres.934348 31928053

[B6] Brooks S. P. Dunnett S. B. (2009). Tests to assess motor phenotype in mice: a user’s guide. Nat. Rev. Neurosci. 10, 519–529. 10.1038/nrn2652 19513088

[B7] Burgos-Artizzu X. P. Dollar P. Lin D. Anderson D. J. Perona P. (2012). Social behavior recognition in continuous video. Proc. IEEE Comput. Soc. Conf. Comput. Vis. Pattern Recognit. 10, 1322–1329. 10.1109/cvpr.2012.6247817

[B8] Carter B. Justin H. S. Gulick D. Gamsby J. J. (2021). The molecular clock and neurodegenerative disease: a stressful time. Front. Mol. Biosci. 8:644747. 10.3389/fmolb.2021.644747 33889597 PMC8056266

[B9] Cepeda C. Tong X. P. (2018). Huntington’s disease: from basic science to therapeutics. CNS Neurosci. Ther. 24, 247–249. 10.1111/cns.12841 29582586 PMC6489829

[B10] Corrochano S. Blanco G. Williams D. Wettstein J. Simon M. Kumar S.. (2018). A genetic modifier suggests that endurance exercise exacerbates Huntington’s disease. Hum. Mol. Genet. 27, 1723–1731. 10.1093/hmg/ddy077 29509900 PMC5932560

[B11] Corrochano S. Renna M. Osborne G. Carter S. Stewart M. May J.. (2014). Reducing Igf-1r levels leads to paradoxical and sexually dimorphic effects in HD mice. PLoS One 9:e105595. 10.1371/journal.pone.0105595 25140802 PMC4139380

[B18] Costall B. Eniojukan J. F. Naylor R. J. (1982). Spontaneous climbing behaviour of mice, its measurement and dopaminergic involvement. Eur. J. Pharmacol. 85, 125–132. 10.1016/0014-2999(82)90457-5 7151866

[B12] d’Isa R. Gerlai R. (2023). Designing animal-friendly behavioral tests for neuroscience research: The importance of an ethological approach. Front. Behav. Neurosci. 16:528. 10.3389/fnbeh.2022.1090248 36703720 PMC9871504

[B13] Dutta D. Majumder M. Paidi R. K. Pahan K. (2021). Alleviation of Huntington pathology in mice by oral administration of food additive glyceryl tribenzoate. Neurobiol. Dis. 153:105318. 10.1016/j.nbd.2021.105318 33636386 PMC8026693

[B14] Fan R. E. Chang K. W. Hsieh C. J. Wang X. R. Lin C. J. (2008). LIBLINEAR: a library for large linear classification. J. Mach. Learn. Res. 9, 1871–1874. 10.1021/ci100073w 21207929

[B15] Ferrante R. J. (2009). Mouse models of Huntington’s disease and methodological considerations for therapeutic trials. Biochim. Biophys. Acta Mol. Basis Dis. 1792, 506–520. 10.1016/j.bbadis.2009.04.001 19362590 PMC2693467

[B16] Hobson L. Bains R. S. Greenaway S. Wells S. Nolan P. M. (2020). Phenotyping in mice using continuous home cage monitoring and ultrasonic vocalization recordings. Curr. Protoc. Mouse Biol. 10:e80. 10.1002/cpmo.80 32813317

[B17] Jin J. Albertz J. Guo Z. Peng Q. Rudow G. Troncoso J. C.. (2014). Neuroprotective effects of PPAR-γ agonist rosiglitazone in N171-82Q mouse model of Huntington’s disease. J. Neurochemm. 125, 410–419. 10.1111/jnc.12190 PMC3642978 23373812

[B19] Kaffman A. White J. D. Wei L. Johnson F. K. Krystal J. H. (2019). Enhancing the utility of preclinical research in neuropsychiatry drug development. Methods Mol. Biol. 2011, 3–22. 10.1007/978-1-4939-9554-7_1 31273690 PMC6895673

[B20] Karl Kieburtz J. Penney B. Peter C. (1996). Unified Huntington’s disease rating scale: reliability and consistency. Mov. Disord. 11, 136–142. 10.1002/mds.870110204 8684382

[B21] Kim A. Lalonde K. Truesdell A. Welter P. G. Brocardo P. S. Rosenstock T. R.. (2021). New avenues for the treatment of huntington’s disease. Int. J. Mol. Sci. 22:8363. 10.3390/ijms22168363 34445070 PMC8394361

[B22] Kumar A. Kumar Singh S. Kumar V. Kumar D. Agarwal S. Rana M. K. (2015). Huntington’s disease: an update of therapeutic strategies. Gene 556, 91–97. 10.1016/j.gene.2014.11.022 25447911

[B23] Kwon D. (2021). Failure of genetic therapies for Huntington’s devastates community. Nature 593:180. 10.1038/d41586-021-01177-7 33963316

[B25] Lana-Elola E. Cater H. Watson-Scales S. Greenaway S. Müller-Winkler J. Gibbins D.. (2021). Comprehensive phenotypic analysis of the dp1tyb mouse strain reveals a broad range of down syndrome-related phenotypes. Dis. Model. Mech. 14:dmm049157. 10.1242/dmm.049157 34477842 PMC8543064

[B26] Levine M. S. Cepeda C. Hickey M. A. Fleming S. M. Chesselet M. F. (2004). Genetic mouse models of Huntington’s and Parkinson’s diseases: illuminating but imperfect. Trends Neurosci. 27, 691–697. 10.1016/j.tins.2004.08.008 15474170

[B27] Mandillo S. Heise I. Garbugino L. Tocchini-Valentini G. P. Giuliani A. Wells S.. (2014). Early motor deficits in mouse disease models are reliably uncovered using an automated home-cage wheel-running system: a cross-laboratory validation. Dis. Model. Mech. 7, 397–407. 10.1242/dmm.013946 24423792 PMC3944499

[B28] McIlwain K. L. Merriweather M. Y. Yuva-Paylor L. A. Paylor R. (2001). The use of behavioral test batteries: effects of training history. Physiol. Behav. 73, 705–717. 10.1016/s0031-9384(01)00528-5 11566205

[B29] Menalled L. B. Kudwa A. E. Miller S. Fitzpatrick J. Watson-Johnson J. Keating N.. (2012). Comprehensive behavioral and molecular characterization of a new knock-in mouse model of Huntington’s disease: ZQ175. PLoS One 7:e49838. 10.1371/journal.pone.0049838 23284626 PMC3527464

[B30] Mingrone A. Kaffman A. Kaffman A. (2020). The promise of automated home-cage monitoring in improving translational utility of psychiatric research in rodents. Front. Neurosci. 14:618593. 10.3389/fnins.2020.618593 33390898 PMC7773806

[B31] Morton A. J. Wood N. I. Hastings M. H. Hurelbrink C. Barker R. A. Maywood E. S. (2005). Disintegration of the sleep-wake cycle and circadian timing in Huntington’s disease. J. Neurosci. 25, 157–163. 10.1523/JNEUROSCI.3842-04.2005 15634777 PMC6725210

[B32] Nakamura T. J. Nakamura W. Yamazaki S. Kudo T. Cutler T. Colwell C. S.. (2011). Age-related decline in circadian output. J. Neurosci. 31:10201. 10.1523/JNEUROSCI.0451-11.2011 21752996 PMC3155746

[B33] Nevison C. M. Hurst J. L. Barnard C. J. (1999). Why do male ICR(CD-1) mice perform bar-related (stereotypic) behaviour? Behav. Processes 47, 95–111. 10.1016/s0376-6357(99)00053-4 24896933

[B34] Pallier P. N. Maywood E. S. Zheng Z. Chesham J. E. Inyushkin A. N. Dyball R.. (2007). Pharmacological imposition of sleep slows cognitive decline and reverses dysregulation of circadian gene expression in a transgenic mouse model of Huntington’s disease. J. Neurosci. 27:7869. 10.1523/JNEUROSCI.0649-07.2007 17634381 PMC6672877

[B35] Palmiter R. D. (2008). Dopamine signaling in the dorsal striatum is essential for motivated behaviors: lessons from dopamine-deficient mice. Ann. N. Y. Acad. Sci. 1129, 35–46. 10.1196/annals.1417.003 18591467 PMC2720267

[B36] Paylor R. Spencer C. M. Yuva-Paylor L. A. Pieke-Dahl S. (2006). The use of behavioral test batteries, II: effect of test interval. Physiol. Behav. 87, 95–102. 10.1016/j.physbeh.2005.09.002 16197969

[B37] Preisig D. F. Kulic L. Krüger M. Wirth F. McAfoose J. Späni C.. (2016). High-speed video gait analysis reveals early and characteristic locomotor phenotypes in mouse models of neurodegenerative movement disorders. Behav. Brain Res. 311, 340–353. 10.1016/j.bbr.2016.04.044 27233823

[B38] Reilmann R. Leavitt B. R. Ross C. A. (2014). Diagnostic criteria for Huntington’s disease based on natural history. Mov. Disord. 29, 1335–1341. 10.1002/mds.26011 25164527

[B39] Schilling G. Becher M. W. Sharp A. H. Jinnah H. A. Duan K. Kotzuk J. A.. (1999). Intranuclear inclusions and neuritic aggregates in transgenic mice expressing a mutant N-terminal fragment of huntingtin. Hum. Mol. Genet. 8, 397–407. 10.1002/mds.26011 9949199

[B40] Tucci V. Achilli F. Blanco G. Lad H. V. Wells S. Godinho S.. (2007). Reaching and grasping phenotypes in the mouse (*Mus musculus*): a characterization of inbred strains and mutant lines. Neuroscience 147, 573–582. 10.1016/j.neuroscience.2007.04.034 17574766

[B41] Voikar V. Gaburro S. (2020). Three pillars of automated home-cage phenotyping of mice: novel findings, refinement and reproducibility based on literature and experience. Front. Behav. Neurosci. 14:575434. 10.3389/fnbeh.2020.575434 33192366 PMC7662686

[B42] Wang H.-B. Loh D. H. Whittaker D. S. Cutler T. Howland D. Colwell C. S. (2018). Time-restricted feeding improves circadian dysfunction as well as motor symptoms in the Q175 mouse model of Huntington’s disease. eNeuro 5:ENEURO.0431–17.2017. 10.1523/ENEURO.0431-17.2017 29302618 PMC5752678

[B43] Werdann M. Zhang Y. (2020). Circadian rhythm and neurodegenerative disorders. Brain Sci. Adv. 6, 71–80. 10.26599/bsa.2020.9050006

[B44] Wiatr K. Szlachcic W. J. Trzeciak M. Figlerowicz M. Figiel M. (2018). Huntington disease as a neurodevelopmental disorder and early signs of the disease in stem cells. Mol. Neurobiol. 55, 3351–3371. 10.1007/s12035-017-0477-7 28497201 PMC5842500

[B45] Yanai S. Endo S. (2021). Functional aging in male C57BL/6J mice across the life-span: a systematic behavioral analysis of motor, emotional and memory function to define an aging phenotype. Front. Aging Neurosci. 13:697621. 10.3389/fnagi.2021.697621 34408644 PMC8365336

[B24] Yeffet L. Wolf L. (2009). “Local trinary patterns for human action recognition,” in Proceedings of the IEEE 12th International Conference on Computer Vision (Piscataway: IEEE Press), 492–497.

[B46] Zhang H. Lecker I. Collymore C. Dokova A. Pham M. C. Rosen S. F.. (2021). Cage-lid hanging behavior as a translationally relevant measure of pain in mice. Pain 162, 1416–1425. 10.1097/j.pain.0000000000002127 33230005 PMC8054539

