# Supplementary material for: Longitudinal home-cage automated assessment of climbing behavior shows sexual dimorphism and aging-related decrease in C57BL/6J healthy mice and allows early detection of motor impairment in the N171-82Q mouse model of Huntington’s disease
Source: Front Behav Neurosci. 2023 Mar 22;17:1148172. doi: 10.3389/fnbeh.2023.1148172 (PMC10073658; doi:10.3389/fnbeh.2023.1148172)
Supplement: Supplementary file 2 [file Data_Sheet_2.PDF]

Supplementary Figures

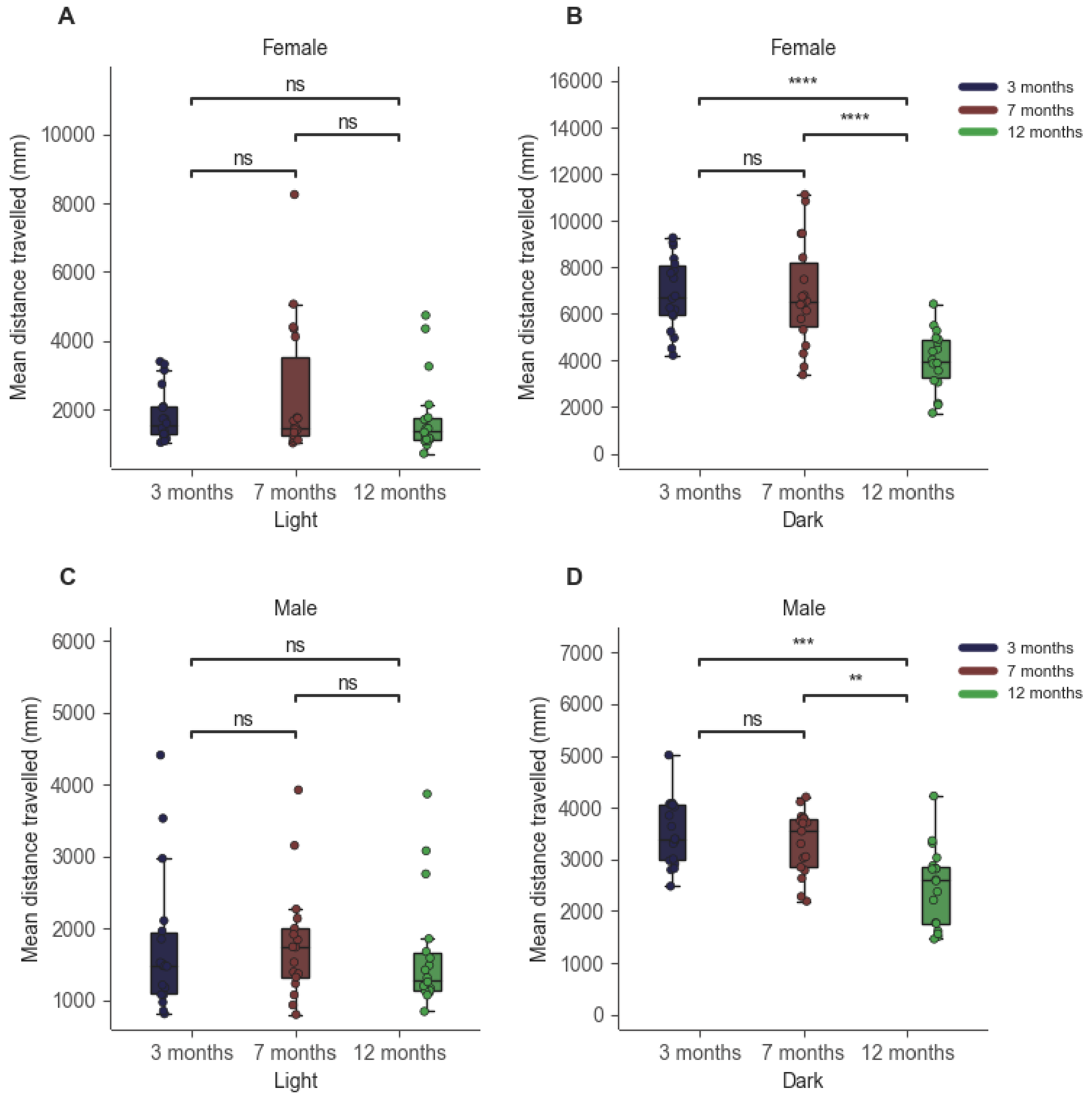

Supplementary Figure 1. Effect of Age on Distance Travelled in C57BL/6J mice. (A) Boxplot of mean distance travelled during light phase in female-only cages split by age. Distance travelled within light phase was averaged for each cage, per day of recording. Three data points per cage (three days of recording per age group) were modelled using a linear mixed-effects model to account for repeated-measures and least-squares means estimated to return adjusted p values of levels of factor combinations. **(B)** Same as A but for dark phase. **(C,D)**. Same as **(A,B)** but for male-only cages in light and dark phase, respectively. **p<0.01, ***p<0.001, ****p<0.0001

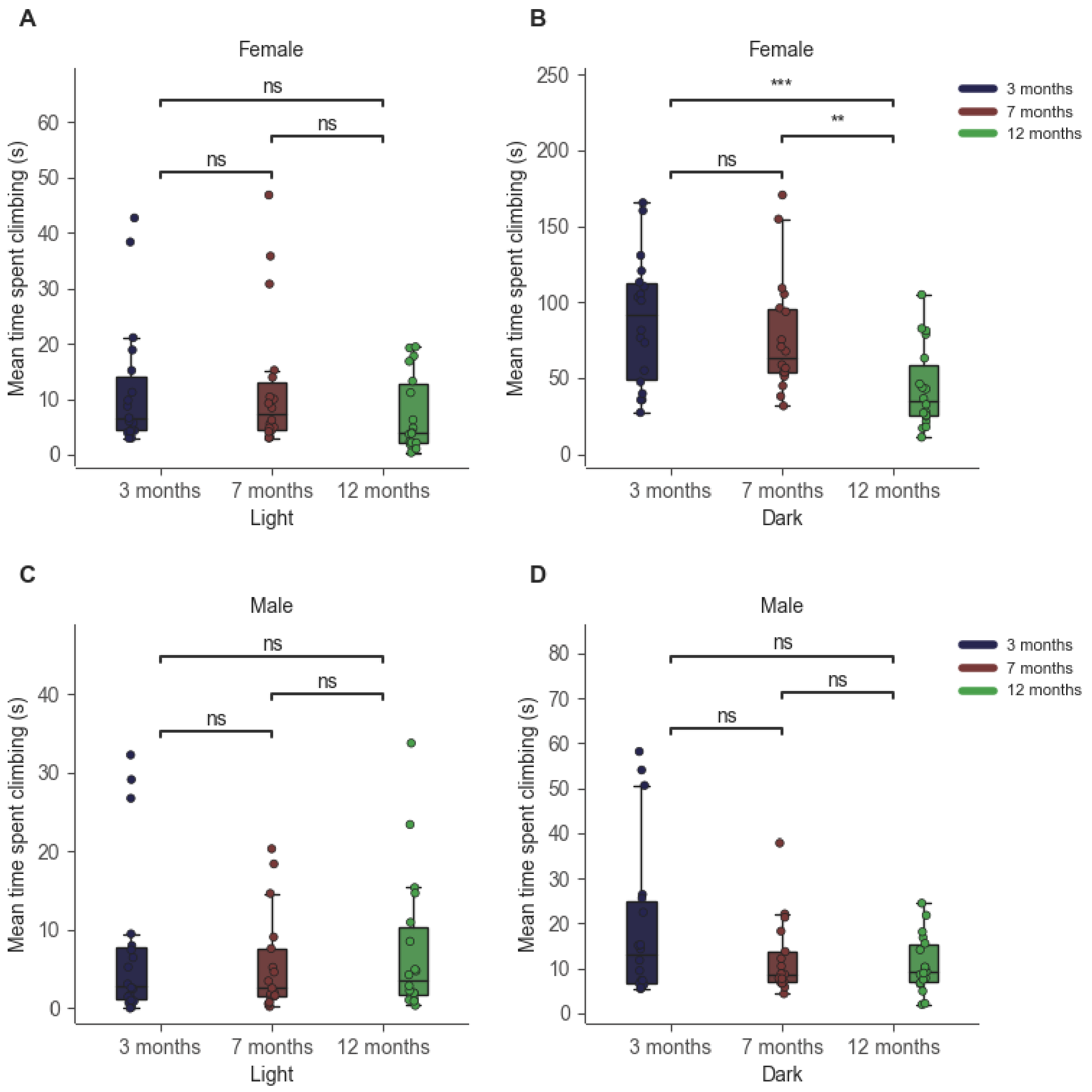

Supplementary Figure 2. Effect of Age on Time Spent Climbing in C57BL/6J mice. (A) Boxplot of mean time spent climbing during light phase in female-only cages split by age. Time spent climbing within light phase was averaged for each cage, per day of recording. Three data points per cage (three days of recording per age group) were modelled using a linear mixed-effects model to account for repeated-measures and least-squares means estimated to return adjusted p values of levels of factor combinations. **(B)** Same as A but for dark phase. **(C,D)**. Same as **(A,B)** but for male-only cages in light and dark phase, respectively. **$p<0.01$, ***$p<0.001$

	Sex					
	Female			Male		
Age	8 Week	13 week	15-16wee	8 Week	13 week	15-16week
WT	7	7	7	8	8	8
Hemi	11	7	3	9	6	5

Supplementary Table 1. N numbers for HD study, where the experimental unit is one individually ventilated cage housing 3 mice.

The model used in this study, N171-82Q, is a fast-progressing model, with weight loss starting at 8 weeks of age and a shortened life span of approximately 24 weeks (with mortality seen as early as 15-16 weeks in some animals) [1]. Some mice carrying the mutation reached humane end point sooner than the other animals in the study, as there was no scientific need for the animals to suffer for further data collection, these animals were humanely sacrificed.

[1] Schilling *et al.,* 1999; https://doi.org/10.1093/hmg/8.3.397
